# Saudi Oncology Society clinical management guidelines for renal cell carcinoma

**DOI:** 10.4103/0974-7796.78548

**Published:** 2011-03

**Authors:** Shouki Bazarbashi, Khaled Al Othman, Mohammed Al Otaibi, Ashraf Abusamra, Danny Rabah, Ali Aljubran, Esam Murshid, Ibraheem Al Oraifi, Mohammed El-naghi, Yasser Bahader, Hussein Soudy, Amjad Rehman

**Affiliations:** Oncology Centre, King Faisal Specialist Hospital and Research Centre, Riyadh, Saudi Arabia; 1 Department of Urology, King Faisal Specialist Hospital and Research Centre, Riyadh; 2 Department of Urology, King Khalid National Guard Hospital and Princess Norah Oncology Center, King Abdulaziz Medical City, Jeddah; 3 Department of Urology, King Khaled University Hospital, King Saud University, Riyadh; 4 Department of Oncology, Riyadh Armed Forced Hospital, Riyadh; 5 Department of Urology, King Fahad Specialist Hospital, Dammam; 6 Department of Oncology, National Guard Hospital, King Abdulaziz Medical City, Riyadh; 7 Department of Medicine, King Abdulaziz University Hospital, Jeddah; 8 Department of Medicine, Aseer Central Hospital, Abha, Saudi Arabia

**Keywords:** Guidelines, genitourinary, Saudi Arabia

## Abstract

In this report, guidelines for the evaluation, medical and surgical management of renal cell carcinoma is presented. It is categorized according to the stage of the disease using the tumor node metastasis staging system, 7^th^ edition. The recommendations are presented with supporting evidence level.

## INTRODUCTION

Renal cancer represents the third common genitourinary cancer in Saudi Arabia after urinary bladder and prostate.[1] It accounts for 2.8% of all male cancers and 1.9% of all female cancers. In 2006, a total of 111 cases where diagnosed in males and 78 cases in females. The Age Standardized Rate in males was 2.4 per 100,000 and in females was 1.5 per 100,000 populations.

All cases of renal cell carcinoma should preferably be seen or discussed in a multidisciplinary forum

## 1. PRE-TREATMENT EVALUATION

**Table:** 

1.1.	Evaluation of suspicious renal cancer:
1.1.1.	History and physical examination
1.1.2.	Blood count, renal and hepatic profile
1.1.3.	CT scan of chest, abdomen and pelvis
1.1.4.	Urine analysis
1.1.5	Urine cytology if suspicious urothelial cancer
1.1.6.	Kidney biopsy is not indicated except in selected cases
1.1.7.	CT brain and bone scan only if clinically indicated

## 2. STAGING[2]

The American Joint Commission on Cancer Staging TNM 7^th^ edition was used

## 3. RISK STRATIFICATION FOR METASTATIC RCC

The Memorial Sloan Kettering cancer center risk classification for metastatic disease will be used:[3] Risk factors are:

**Table:** 

3.1.	A Karnofsky performance status (KPS) of <80%
3.2.	Serum lactic dehydrogenase (LDH) level >1.5 times the upper limit of normal
3.3.	Corrected serum calcium >10 mg/dL (2.5 mmol/L)
3.4.	Hemoglobin concentration below the lower limit of normal
3.5.	No prior nephrectomy (i.e., no disease-free interval)

Each of the above gives a score of one. Patients will be classified according to the total score as follows:

**Table:** 

0	points	Low risk
1, 2	points	Intermediate risk
3, 4, 5	points	High risk

## 4. TREATMENT

**Table:** 

4.1.	Localized disease (stage I-III): treatment is surgical excision. The following should be considered for surgery:
4.1.1.	Nephron sparing surgery is indicated if surgically possible in:
4.1.1.1.	Tumor less than 4 cm (EL-1)
4.1.1.2.	Bilateral disease
4.1.1.3.	Solitary kidney (anatomic or functional)
4.1.1.4.	Patients at high risk for recurrent RCC (e.g. Von Hippel-Lindau syndrome)
4.1.2.	Radical nephrectomy both open or laparoscopic are acceptable, however laparoscopic is preferable in experienced centers (EL-1)
4.1.3.	Lymph node dissection is not indicated. Clinically resectable enlarged lymph nodes should be removed at the time of nephrectomy (EL-3)
4.1.4.	Adrenal gland can be spared except in large upper pole tumors (EL-3)
4.1.5.	No adjuvant therapy is of known benefit in complete resection (EL-1)
4.1.6.	Follow up: No standard follow-up protocol is recommended.
4.2.	Metastatic/advanced unresectable disease: several scenarios are possible and should be considered:
4.2.1.	Potentially resectable primary with solitary metastasis or multiple resectable lung metastasis: those patients should undergo primary nephrectomy and resection of the metastatic lesion/s (EL-2).[4] Following complete resection no further therapy is indicated (EL-3).
4.2.2.	Potentially resectable primary and multiple metastasis: those patients should undergo resection of the primary tumor if in good performance status (EL-1),[5,6] then should start systemic therapy as follows:
4.2.2.1.	Clear cell histology, good and intermediate risk: options are Sunitinib[7] (EL-1), Bevacizumab and Interferon a-2a[8,9] or pazopanib[10] (EL-1).
4.2.2.2.	Clear cell histology and poor risk: Temsirolimus[11] (EL-1)
4.2.2.3.	Non-clear cell histology: Temsirolimus (EL-2)[12] or Sunitinib[13] (EL-2), or Sorafenib[14] (EL-2). Medullary and collecting duct carcinoma should be treated with platinum-based chemotherapy[15,16] (EL-3)
4.2.3.	Unresectable primary with or without metastatic disease: those patients with good performance status should be offered the systemic therapy as in Item 4.2.2
4.2.3.1.	Recurrent disease post-primary nephrectomy:
4.2.3.2.	Resectable solitary metastasis: surgical resection should be attempted[17–19] (EL-2). No systemic therapy is of benefit following complete resection (EL-3).
4.2.3.3.	Non-resectable recurrence: treat as in Item 4.2.2
4.2.4.	Second-line therapy post-TKI failure: Everolimus (EL-1)[20,21]
